# Characteristics of Gut Microbiota in Small for Gestational Age Infants with Very Low Birth Weight

**DOI:** 10.3390/nu14235158

**Published:** 2022-12-04

**Authors:** Hung-Yang Chang, Jen-Shiu Chiang Chiau, Jui-Hsing Chang, Chyong-Hsin Hsu, Chia-Ying Lin, Mary Hsin-Ju Ko, Hung-Chang Lee

**Affiliations:** 1 Department of Pediatrics, MacKay Children’s Hospital, Taipei 104, Taiwan; 2 Department of Medicine, MacKay Medical College, New Taipei City 252, Taiwan; 3 Department of Medical Research, MacKay Memorial Hospital, Taipei 104, Taiwan; 4 Department of Pediatrics, Hsinchu MacKay Memorial Hospital, Hsinchu 300, Taiwan

**Keywords:** small for gestational age, intrauterine growth restriction, microbiota, preterm, very low birth weight infants

## Abstract

Small for gestational age (SGA) birth is associated with high rates of mortality and morbidity in preterm infants. The aim of this preliminary observational study was to investigate the difference in gut microbiota between SGA and appropriate for gestational age (AGA) preterm infants with very low birth weight (VLBW). We included 20 VLBW preterm infants (SGA, *n* = 10; AGA, *n* = 10) in this study. Stool samples were collected on days 7, 14, and 30 after birth. We performed 16S ribosomal DNA sequencing to compare microbiota composition between both groups. The SGA group exhibited a lower abundance of *Klebsiella* on day 14 (SGA, 0.57%; AGA, 7.42%; *p* = 0.037). On day 30, the SGA group exhibited a lower abundance of *Klebsiella* (SGA 3.76% vs. AGA 16.05%; *p* = 0.07) and *Enterobacter* (SGA 5.09% vs. AGA 27.25%; *p* = 0.011) than the AGA group. Beta diversity demonstrated a separation of the bacterial community structure between both groups on day 30 (*p* = 0.019). The present study revealed that a distinct gut microbiota profile gradually develops in SGA preterm infants with VLBW during the early days of life. The role of changes in gut microbiota structure warrants further investigation.

## 1. Introduction

Small for gestational age (SGA) infants are broadly defined as newborns with birth weight (BW) below the 10th percentile or two standard deviations of body weight of newborns of the same gestational age (GA) and gender [1]. Although the terms “SGA” and “intrauterine growth restriction” (IUGR) have been used synonymously in the literature, the conditions exhibit clear differences [2]. IUGR is commonly defined as a reduction in fetal growth rate caused by pathophysiological events. Moreover, an infant can be described as an SGA after measuring the BW postnatally. Not all newborns who are SGA have IUGR, and not all fetuses who have IUGR are SGA. SGA birth is attributed to a complex interaction of multiple perinatal factors, including pregnancy-induced hypertension, age of the mother, socioeconomic status, obesity, smoking, and placental abnormalities [2,3]. SGA preterm birth is associated not only with high morbidity and mortality rates in infants, but also with metabolic syndromes, cardiovascular diseases, and neurodevelopmental impairments in later stages of life [4,5,6,7]. Many studies have linked these long-term health outcomes with microbiota in early life [8,9].

The gastrointestinal tract is colonized by a complex and diverse microbial community, and the microbial colonization process is influenced by various host and environmental factors, including GA, mode of delivery, feeding strategy, probiotic administration, immediate surroundings (e.g., neonatal intensive care unit for infants), and medical interventions [10,11]. The gut microbiome is crucial for nutrient absorption, energy storage, and metabolism modulation [12]; therefore, the disruption of gut microbiota alters nutrient absorption, influences growth and neurodevelopment, and causes metabolic diseases [11,13]. The gut microbiota of preterm newborn infants, compared to that of full-term newborns, is unique because it is characterized by the delayed colonization of common bacteria and an abundance of pathogens [14,15]. Moreover, preterm infants have a high risk of impaired growth, nutrition, and neurodevelopment, especially SGA infants with very low birth weight (VLBW) [4,5].

Previous studies have revealed that intestinal microbiota, diversity, and metabolite profiles of IUGR piglets were significantly different from those of normal weight piglets, suggesting that gut microbes are potentially associated with impaired growth and development [16,17,18,19]. In rats, IUGR induces changes in the intestinal microbiota composition, and these changes persist throughout life [10,20]. However, very few studies have addressed the interplay between gut microbiota and SGA in humans [21,22,23]. Thus, the association between gut microbiota composition and preterm SGA infants should be elucidated.

We hypothesized that the microbial colonization process in SGA preterm infants was different from that in normal-weight preterm infants. Thus, in this preliminary observational study, we investigated whether the gut microbiota composition of SGA preterm infants with VLBW was unique.

## 2. Materials and Methods

### 2.1. Study Participants

The current study was a subgroup analysis of a prospective cohort study including VLBW preterm infants born at MacKay Children’s Hospital and admitted to its neonatal intensive care unit from May 2017 to May 2018. This study was financially supported by a grant (MMH-107-84) from MacKay Memorial Hospital. The sampling protocol and research proposal were approved by the Institutional Review Board of MacKay Memorial Hospital (IRB number: 17MMHIS026e). Written informed consent was obtained from parents or guardians of the infants.

In this study, we included VLBW preterm infants with GA ≤ 32 weeks. The exclusion criteria were as follows: infants with major congenital anomalies or malformations, no administration of nutrients or liquid by mouth for ≥3 d, probiotic use during the study period, and antibiotic use after the age of 7 d. The enrolled infants were divided into two groups: those whose BW was <10th percentile of that of the same GA (SGA group) and those whose BW was appropriate for their GA (AGA group). The definition of SGA or AGA in this study was a criterion derived from a nationwide birth weight percentile by gestational age study conducted in Taiwan [24]. All infants were fed with their mother’s fresh or frozen breast milk without pasteurization. The following maternal and neonatal information was obtained from the medical records: maternal age, preeclampsia (blood pressure > 140/90 mmHg after 20 weeks of gestation with significant proteinuria), antenatal steroid treatment, mode of delivery, GA, BW, postnatal age, sex, respiratory distress syndrome, chronic lung disease (oxygen dependent at GA of 36 weeks), retinopathy of prematurity, duration of hospital stays, and age and body weight at discharge.

### 2.2. Sample Collection, Microbial DNA Extraction, PCR Amplification, and Sequencing

Fresh feces were collected from the diapers of the participants by medical staff at three time-points: 7, 14, and 30 d. All samples were immediately stored at −80 °C until DNA extraction. Microbial DNA extraction and microbial community composition analysis were performed as previously described [10,25]. Briefly, fecal DNA was extracted using a QIAmp^®^ DNA stool mini kit (Qiagen, Hilden, Germany) according to the manufacturer’s instructions. The V3-V4 region of the 16S rRNA gene was amplified by PCR, using universal primers (16S Amplicon PCR Forward Primer: 5′ TCGTCGGCAGCGTCAGATGTGTATAAGAGACAGCCTACGGGNGGCWGCAG; 16S Amplicon PCR Reverse Primer: 5′ GTCTCGTGGGCTCGGAGATGTGTATAAGAGACAGGACTACHVGGGTATCTAATCC). DNA was quantified using a NanoDrop ND2000 spectrophotometer (Thermo Scientific, Waltham, MA, USA), Qubit dsDNA HS Assay Kit (Invitrogen, Waltham, MA, USA), and Labchip GX Touch 24 (Applied Biosystems, Waltham, MA, USA). The amplicons were then pooled in equimolar quantities and paired-end sequencing (2 × 300 bp) was performed on an Illumina MiSeq platform according to standard protocols.

### 2.3. Bioinformatics Analysis of Microbiota Composition

We analyzed the quality of the obtained raw sequence reads using Quantitative Insights into Microbial Ecology (QIIME). QIIME provided operational taxonomic unit tables containing the microbiome composition and relative abundance for each bacterial taxa (from phylum to genus) for each sample. Alpha diversity was measured using the Chao1 and Shannon indices. Principal coordinate analysis (PCoA) using “vegan” in the R package was applied to visualize the dissimilarity of bacterial populations (beta diversity) between the SGA and AGA groups.

### 2.4. Statistical Analyses

Patient characteristics were assessed using a two-sample t-test for continuous variables and Fisher exact test for categorical variables. Microbial composition and alpha diversity were compared between the two groups at three time-points using the Mann–Whitney U test. To determine significant differences over time within a group, the Kruskal–Wallis test was conducted for multiple comparisons, followed by the post-hoc Dunn’s test using Benjamini–Hochberg for correction. Permutational multivariate analysis of variance (PERMANOVA) with the Adonis function available in the “vegan” package of R was used to assess the effects of relevant confounding factors, including preeclampsia and GA. Spearman’s rank correlation test was used to evaluate the association between the bacterial abundance and growth rate. Differences were considered statistically significant at *p* < 0.05.

## 3. Results

### 3.1. Demographic Characteristics

Initially in this study, 81 breastfed VLBW infants with GA ≤ 32 weeks admitted to our NICU were included. However, after excluding infants who were administered probiotics (*n* = 48) and those with missing stool samples (*n* = 13), only the remaining 20 patients were included in the final analysis.

Based on BW, the enrolled VLBW infants were divided into the SGA (*n* = 10) and AGA (*n* = 10) groups. Mean GA and BW were 29.2 ± 0.9 weeks and 1310 ± 130 g in the AGA group, and 29.6 ± 2.5 weeks and 1079 ± 278 g in the SGA group, respectively. At the time of discharge, mean GA and BW were 37.3 ± 3.5 weeks and 2574 ± 380 g in the AGA group and 38.2 ± 1.9 weeks and 2389 ± 108 g in the SGA group, respectively. Nine infants from the SGA group were SGA even at the time of discharge, but none of the patients from the AGA group were SGA at the time of discharge. BW and body weight at the time of discharge were significantly lower in the SGA group than that in the AGA group. According to our Unit’s protocol, all infants received prophylactic antibiotics after birth. All infants only received a 3-day course of antibiotics because the blood culture results revealed no growth. The incidence of maternal preeclampsia was significantly higher in the SGA group than that in the AGA group. No statistically significant differences were observed in other demographic and clinical characteristics between the two groups (Table 1).

### 3.2. Analysis of Microbiota Composition

**Figure 1:**
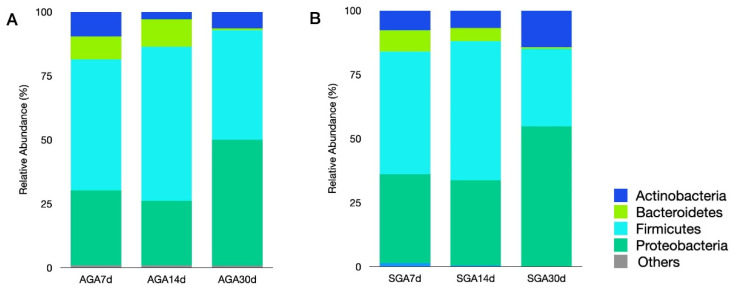
Relative abundance of dominant bacterial phyla in the AGA (**A**) and SGA groups (**B**) on days 7, 14, and 30. Abbreviations: AGA, appropriate for gestational age; SGA, small for gestational age.

Sequence data that passed quality control were used to generate 60 samples, i.e., samples from the 20 patients collected at three different time-points. The overall microbial composition at the phylum level is presented in Figure 1. Four phyla (*Actinobacteria, Bacteroidetes, Firmicutes, and Proteobacteria*) were dominant across all samples in both groups. *Firmicutes* was the dominant phylum in both groups during the first 2 weeks (48–54% in the SGA group and 51–60% in the AGA group). *Proteobacteria* was the most abundant phylum in day 30 samples of both groups (54.8% in the SGA group and 49.2% in the AGA group). The abundance of *Bacteroidetes* decreased over time and accounted for a small proportion in day 30 samples of both groups (0.62% in the SGA group and 0.72% in the AGA group). Predominant phyla were similar in the AGA and SGA groups; moreover, the abundance of bacterial phyla was also similar.

**Figure 2:**
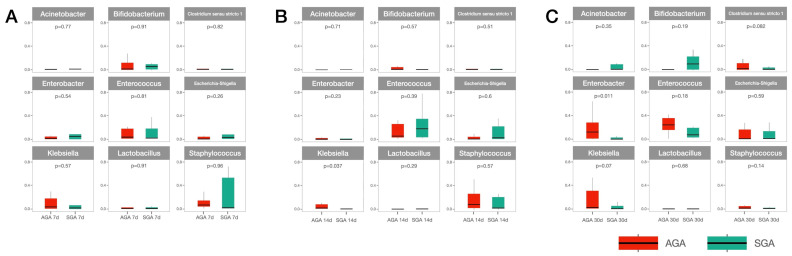
Relative abundance of the dominant bacterial genera in the AGA and SGA groups. Samples collected on days (**A**) 7, (**B**) 14, and (**C**) 30. Abbreviations: AGA, appropriate for gestational age; SGA, small for gestational age.

The main microbial composition at the genus level is presented in Figure 2. *Enterococcus*, *Staphylococcus, Escherichia/Shigella, Bifidobacterium, Enterobacter, and Acinetobacter* were the main genera in both groups at all time-points. The SGA group exhibited a lower abundance of *Klebsiella* on days 14 (Figure 2B; SGA 0.57% vs. AGA 7.42%; *p* = 0.037) and 30 (Figure 2C; SGA 3.76% vs. AGA 16.05%; *p* = 0.07). On day 30, the SGA group exhibited a significantly lower abundance of *Enterobacter* spp. (Figure 2C; SGA 5.09% vs. AGA 27.25%; *p* = 0.011) than the AGA group. The differences remained significant after adjusting for preeclampsia and GA. The relative abundance of *Klebsiella* on days 14 and 30 and that of *Enterobacter* on day 30 was not related to growth rate when samples from data from both groups was collectively assessed (r = −0.09, *p* = 0.73; r = −0.003, *p* = 0.99; and r = −0.033, *p* = 0.89, respectively). No statistically significant difference at other genus level was observed between the two groups at any time-point.

Comparative analysis of microbial changes within each group revealed that the abundance of *Bacteroides* was significantly decreased on days 14 and 30 (0.08% and 0.41%, respectively) compared to that on day 7 (1.26%, *p* = 0.04) in the SGA group. Other major bacterial genera exhibited no statistically significant differences over time within the group. Although the proportion decreased over time, *Staphylococcus* was the most abundant genus in both groups on day 7 (Figure 2A; SGA group: 31.1%, 15.9%, and 1.7% on days 7, 14, and 30, respectively; AGA group: 26.5%, 15.8%, and 6.2% on days 7, 14, and 30, respectively). *Enterococcus* was the most abundant genus in both groups on day 14 (Figure 2B; SGA group: 9.5%, 27.1%, and 13.5% on days 7, 14, and 30, respectively; AGA group: 16.0%, 32.9%, and 16.0% on days 7, 14, and 30, respectively). Although all infants were fed breast milk, the abundance of genus *Bifidobacterium* did not increase over time in either group (SGA group: 6.9%, 6.0%, and 12.9% on days 7, 14, and 30, respectively; AGA group: 7.5%, 2.4%, and 5.4% on days 7, 14, and 30, respectively). The abundance of *Lactobacillus* was also very low in both groups (SGA group: 1.9%, 0.2%, and 0.1% on days 7, 14, and 30, respectively; AGA group: 2.1%, 0.5%, and 1.1% on days 7, 14, and 30, respectively).

### 3.3. Diversity Analysis of Gut Microbiota

**Figure 3:**
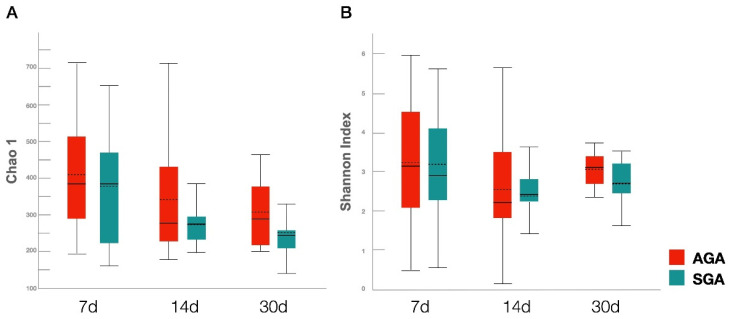
Comparison of gut microbial species richness and diversity in the AGA and SGA groups on days 7, 14, and 30. (**A**) Chao1 estimator. (**B**) Shannon index. Boxes, lines, and whiskers represent the interquartile range, median, and range, respectively. Abbreviations: AGA, appropriate for gestational age; SGA, small for gestational age.

We observed no significant differences in bacterial alpha diversity, measured by the Chao1 and Shannon indices, between the SGA and AGA groups across all time-points. Moreover, no significant difference was observed in alpha diversity at different time-points within a group. (Figure 3).

**Figure 4:**
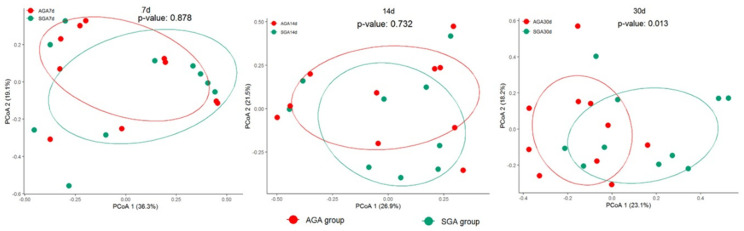
PCoA of microbial communities in the AGA and SGA groups on days 7, 14, and 30. Abbreviations: PCoA, principal coordinate analysis; AGA, appropriate for gestational age; SGA, small for gestational age.

Despite the overlap, the overall community structure was significantly different between the SGA and AGA groups on day 30 (*p* = 0.013) (Figure 4). However, the microbial community structure was relatively similar between the AGA and SGA groups on days 7 (*p* = 0.878) and 14 (*p* = 0.732).

## 4. Discussion

In this observational pilot study, we revealed differences in the gut microbial composition between SGA and AGA preterm infants at 1 month of age. These results imply that SGA perturbs the development of the gut microbiota gradually in VLBW preterm infants. SGA are complex processes that are highly related to maternal and placental conditions. The maternal microbiota plays an important role in the development and growth of the fetus [26]. Potential determinants of the maternal microbiota composition include preterm rupture of membranes, chorioamnionitis, preeclampsia, and antibiotic use. Although there was no difference in maternal antibiotic use in both groups, different prenatal antibiotics spectra, dose, and duration may also affect the infants’ microbiota [27,28,29,30,31]. The placenta and meconium were previously thought to be sterile, and the existence of microbiota was still under debate [32,33]. However, a recent study has shown that the microbiomes of the placenta and meconium were altered in IUGR patients [23]. An increase in the abundance of family *Neisseriaceae* and anaerobic bacteria such as *Desulfovibrio* in the placenta was found, which reflects the emergence of a hypoxic environment in IUGR patients [23]. The microbiome of meconium is also related to the growth of preterm infants [21]. However, in the current study, we could not collect microbiota samples from the placenta. Moreover, the collected meconium samples were insufficient for analysis.

The predominant phyla in VLBW infants were *Actinobacteria, Bacteroidetes, Firmicutes*, and *Proteobacteria*, which is consistent with previous reports [10,34]. Previous studies have reported that IUGR piglets have a higher proportion of phylum *Proteobacteria*, which includes a wide variety of pathogenic bacteria [16,18]. Reduced relative abundance of *Firmicutes* is closely associated with neonates with IUGR [23]. Infants born with IUGR also exhibit a significantly lower *Firmicutes/Bacteroidetes* ratio [23]. However, contrary to the results of previous studies, *Firmicutes* was found to be dominant in the early samples of the current study. Although the difference in the abundance of Proteobacteria was not significant between the two groups, it was the most dominant phylum on day 30 in both groups. Since many potential pathogens and opportunistic microbes belong to the phylum *Proteobacteria*, these findings suggest that potential pathogens might have colonized the gastrointestinal tract of VLBW preterm newborns, making them more susceptible to diseases.

Moreover, in previous studies, several genera have been associated with SGA or IUGR animals/patients; thus, the results at the genus level are not consistent. Increased abundances of *Fusobacterium, Campylobacter, Moryella, Oscillibacter, Escherichia-Shigella*, and *Pasteurella*, and decreased abundances of *Streptococcus, Bacteroides, Clostridium sensu stricto 1*, and *Ruminococcaceae* have been linked with IUGR in piglets [16,18,19]. In humans, increased abundance of *Prevotella* in preterm AGA infants was observed [21]. In the current study, we found lower abundances of *Enterobacter* and *Klebsiella* in the SGA group. However, this result has not been previously reported. Moreover, the causal relationships and underlying mechanisms remain unclear. However, increased abundances of *Enterobacter and Klebsiella* have been linked to subcutaneous fat accumulation and weight gain in animal studies, which may explain the relatively low levels of these two genera in SGA preterm infants [35,36].

In accordance with previous studies, the current study confirmed that Staphylococcus was the most abundant genus among preterm infants [10,37]. Furthermore, human milk contains an oligosaccharide that promotes colonization of *Bifidobacterium* and *Lactobacillus* [38]. The loss of *Lactobacillus* in the placenta may be associated with the development of IUGR [23]. Low proportions of *Lactobacillus* and *Bifidobacterium* have also been found in IUGR piglets [16,18]. Although we did not observe a significant change in the abundance of *Bifidobacterium* and *Lactobacillus* between the two groups, nutrition (including probiotics) could be a useful intervention to alter microbial composition to prevent SGA in piglets or preterm infants [39,40].

A lower diversity of the intestinal microbiota is considered a marker of dysbiosis [8]. Although the alpha diversity in IUGR piglets has been reported to be significantly lower than that in controls [18], a microbiome analysis of humans revealed no difference between IUGR patients and normal participants [23]. In accordance with these results, we did not observe any significant difference in the alpha diversity between the two groups. Previous studies have revealed that the similarity of the microbiome community increased over time in preterm infants, based on beta diversity analysis [10,41]. However, significant separation was only noted on day 30 in the current study. From a relatively sterile environment during pregnancy, parturition transitions the fetus to novel microbial environments. Several postnatal conditions may affect the infant’s microbiome, including birth mode, gestational age, drugs, and nutritional supplements. The role of birth mode on the initial acquisition and subsequent development of the infant microbiome remains controversial [42]. Microbiota of vaginally-delivered infants resemble that of their mother’s vaginal microbiota and are initially enriched in *Lactobacillus*, whereas infants born by cesarean section are exposed to bacteria originating from their mother’s skin and the hospital environment, such as *Staphylococcus* [43,44]. Most VLBW preterm infants are delivered by cesarean section, which may explain why *Staphylococcus* is the most dominant species and the abundance of *Lactobacillus* is relatively low in our study. It is also possible that the results of this study could be confounded by differences between the groups in gestational age. A previous study revealed that the richness and diversity of microbiota in preterm infants were positively associated with postnatal age, not gestational age itself [45]. Drugs commonly used in preterm infants may also interfere with the development of gut microbiota, including antibiotics, prophylactic antifungal treatment, probiotics, and caffeine [10,46]. Nutritional supplements, such as iron, breast milk fortifier, and vitamin D, have also been characterized for their effects on microbiome composition [47]. All these factors may contribute to the gradual development of significant differences in microbial composition between the SGA and AGA groups. Furthermore, animal studies have reported impairment of intestinal development in IUGR piglets [48,49]. Thus, we hypothesized that limited nutrient storage at birth is accompanied by injured intestinal barriers and insufficient growth, which changes the establishment and succession of microbial composition in SGA infants. However, further studies are required to confirm this relationship.

### Limitations

The strengths of our study include the use of serially collected fecal samples to compare the gut microbiota profiles and the relatively homogenous characteristics of both groups. However, this study has some limitations, including the small sample size and relatively short study period. The findings cannot be generalized to all preterm or term infants as participants came from the same hospital. A larger sample size and a longer study duration must be considered in future studies. Finally, the preexistence of a higher risk of preeclampsia in the SGA group created selection bias. A recent review suggested that aberrations in the composition of the microbiome may play a role in the pathogenesis of preeclampsia [50]. Although all confounding factors were not controlled, the most significant in microbiome development, i.e., breastfeeding, was accounted for [44,51]. The influence of the microbiome on compensatory growth or extrauterine growth restriction (EUGR) was not included because the weight of most of the SGA infants was still below the 10th percentile at the time of discharge. Feeding, weight gain, and catch-up growth are related to microbial community structure [10,22,52,53,54,55]. In a rat model, the decrease in the abundance of *Lactobacillus* was significantly associated with catch-up growth [10]. Increased abundance of *Staphylococcus* has been related to SGA at the time of discharge in preterm infants [21]. Li et al. reported that the abundance of genus *Salinivibrio* significantly decreased in VLBW infants with EUGR at the age of 1 month [22]. Although we did not find a relationship between bacterial abundance and growth rate, it is still unclear whether the disturbed gut microbial composition in this study is attributed to SGA or poor growth after birth.

## 5. Conclusions

In conclusion, we demonstrated that SGA preterm infants with VLBW had distinct intestinal microbiota development compared to that of AGA controls. On day 30, SGA infants exhibited lower *Klebsiella* and *Enterobacter* abundances, and the bacteria from this group clustered separately from those of the AGA group. Our results provide initial evidence of potential connections between early development of intestinal microbiota and SGA. Long-term and clinical impacts in a larger group of subjects from several hospitals should be evaluated to address underlying mechanisms of microbial inoculation before and after delivery. Future multicenter clinical studies can then inform novel strategies to address changes in intestinal microbiota in SGA infants.

## Figures and Tables

**Table 1:** Demographics and clinical characteristics of the participants.

	SGA Group ^a^ (*n* = 10)	AGA Group ^a^ (*n* = 10)	*p* Value
Maternal age ^a^	35 (18–39)	32 (25–45)	0.690
Prenatal steroid use	10	9	0.343
Prenatal antibiotics use	3	5	0.388
Preeclampsia	7	1	0.005
Cesarean delivery	7	9	0.290
GA (week) ^a^	29.5 (27–32)	29.0 (27–32)	0.235
BW (g) ^a^	1119 (734–1361)	1270 (1120–1465)	0.034
Male	3	6	0.196
APGAR score at 5 min ^a^	9 (7–10)	9 (7–10)	1.000
Reach full feeding (d) ^a^	21.0 (9.0–44)	17.5 (9–21)	0.709
RDS needs surfactant	0	3	0.081
CLD	3	3	1.000
ROP needs treatment	0	1	0.343
Total hospitalization days ^a^	48.5 (36–77)	51.5 (36–98)	0.715
GA at discharge ^a^	38.0 (36–43)	37.0 (34–44)	0.488
Weight at discharge ^a^	2380 (2260–2582)	2408 (2294–4184)	0.109
Weight less than 10th percentile at discharge	9	0	0.0001

No cases of sepsis, severe intraventricular hemorrhage, patent ductus arteriosus requiring treatment, or necrotizing enterocolitis were observed in either group. Values represent the number of participants, unless otherwise specified. ^a^ Values are expressed as median (IQR). Abbreviations: AGA, appropriate for gestational age; BW, birth weight; CLD, chronic lung disease; GA, gestational age; RDS, respiratory distress syndrome; ROP, retinopathy of prematurity; SGA, small for gestational age.

## Data Availability

All data generated or analyzed during this study are available from the corresponding author on reasonable request.
